# Severe Combined Immunodeficiency Disorder due to a Novel Mutation in Recombination Activation Gene 2: About 2 Cases

**DOI:** 10.1155/2021/8819368

**Published:** 2021-01-07

**Authors:** Ibtihal Benhsaien, Fatima Ailal, Khadija Elazhary, Jalila El bakkouri, Abdallah Badou, Ahmed Aziz Bousfiha

**Affiliations:** ^1^Clinical Immunology Unit, Infectious Disease Department; Children Hospital, IBN Rochd University Hospital, Casablanca, Morocco; ^2^Cellular and Molecular Pathology Laboratory, Faculty of Medicine and Pharmacy of Casablanca, Hassan II University, Casablanca, Morocco; ^3^Clinical Immunology, Autoimmunity and Inflammation Laboratory (LICIA), Faculty of Medicine and Pharmacy of Casablanca, Hassan II University, Casablanca, Morocco; ^4^Immunology Laboratory, IBN Rochd University Hospital, Casablanca, Morocco

## Abstract

Severe combined immunodeficiency (SCID) comprises a heterogeneous group of inherited immunologic disorders with profound defects in cellular and humoral immunity. SCID is the most severe PID and constitutes a pediatric emergency. Affected children are highly susceptible to bacterial, viral, fungal, and opportunistic infections with life-threatening in the absence of hematopoietic stem cell transplantation. We report here two cases of SCID. The first case is a girl diagnosed with SCID at birth based on her family history and lymphocyte subpopulation typing. The second case is a 4-month-old boy with a history of recurrent opportunistic infections, BCGitis, and failure to thrive, and the immunology workup confirms a SCID phenotype. The genetic study in the two cases revealed a novel mutation in the RAG2 gene, c.826G > A (p.Gly276Ser), in a homozygous state. The novel mutation in the RAG2 gene identified in our study may help the early diagnosis of SCID.

## 1. Introduction

Severe combined immunodeficiencies (SCIDs) are a group of inherited disorders responsible for severe dysfunctions of the immune system that lead to the absence or dysfunction of the T and B cells derived from the thymus gland and bone marrow, thus affecting both cellular and humoral adaptive immunity [1]. The incidence and prevalence of SCID vary in different parts of the world and are reported to be higher in countries with a high rate of consanguinity. In Saudi Arabia, the prevalence reported is 2,906 live births [2], which is (20×) higher than the incidence reported from USA NBS programs [3]. This is the most severe disorder among primary immunodeficiency diseases threatening children's life. Actually, seventeen molecular defects are recognized as causing SCID [4]. Depending on the ethnicity, it is estimated that RAG1/2 mutations account for nearly 50% of patients with T-B-NK + SCID [5]. Recombination-activating genes (RAG1/2) contribute to the VDJ (variable, diversity, and joining) recombination which leads to the generation of diverse antigen receptors [6]. Null mutations cause severe combined immunodeficiency (SCID), with the absence of both B and T cells and preserved natural killer (NK) cells (T-B-NK+) [7].

By contrast, hypomorphic RAG mutations that allow residual expression and function of the mutant protein, enabling partial T and B lymphocyte development, may cause a spectrum of phenotypes with prominent immune dysregulations, as observed in patients with Omenn syndrome [8], leaky SCID with a predominance of T-cell receptor (TCR) *γδ*^+^ T cells [9, 10], and combined immunodeficiency with granulomatous disease and/or autoimmunity [11].

## 2. Materials and Methods

### 2.1. Blood Sample Collection

Whole blood was collected in EDTA-precoated test tubes.

### 2.2. Flow Cytometry

Red blood cells were removed from whole blood using an RBC lysis buffer. Cells were then stained with fluorochrome-conjugated anti-human CD3, CD4, CD8, CD19, CD16, and CD56 antibodies. Cells were collected with a FACSCanto II flow cytometer (a FACSCalibur from BD Biosciences, San Jose, CA) and analyzed with the FACSDiva software.

### 2.3. Genetic Study

Genomic DNA was isolated from 300 *µ*L of peripheral blood using Maxwell 16 Blood DNA Purification Kit (Promega, Madison, WI, USA) according to the manufacturer's protocol. Both parents of the patient gave their written and informed consent for the genetic study.

10 ng of DNA was used to amplify the coding region using Ion AmpliSeq™ Inherited Disease Panel (Ion Torrent, Thermo Fisher Scientific) according to the manufacturer's protocol.

The sample was reduced to a final concentration at ∼100 pM. Emulsion PCR was performed using the Ion PGM Hi-Q OT2 KIT (Ion Torrent™, Thermo Fisher Scientific) according to the manufacturer's protocol. Following amplification and recovery, an Ion Sphere Quality Control Kit (Ion Torrent™, Thermo Fisher) was used to evaluate the sample's quality according to the manufacturer's protocol. Next, the enrichment was completed by selectively binding the ISPs containing amplified library fragments to streptavidin-coated magnetic beads, removing empty ISPs through washing steps, and denaturing the sample strands to allow the collection of the positive template ISPs.

Sequencing primer and polymerase were added to the final enriched spheres ISPs prior to loading into an Ion 318 Chip according to the Ion PGM Hi-Q Sequencing Kit (Ion Torrent™, Thermo Fisher Scientific).

Sequencing was carried out on the Personal Genome Material (Ion Torrent™, Thermo Fisher Scientific). For a sequence variant to be considered authentic, sequencing coverage of 250 was used as a minimum requirement in this study. Ion AmpliSeq Inherited Disease Panel targets the following PID genes: IL2RG for X-linked SCIDs, RAG1 for severe combined immunodeficiency, RAG2 for severe combined immunodeficiency, BTK for agammaglobulinemia X-linked type 1, WAS for Wiskott–Aldrich syndrome, and AIRE for autoimmune polyendocrine syndrome.

### 2.4. Data Analysis

Sequence data were processed using the Torrent Suite software v 5.0.4 (Ion Torrent™, Thermo Fisher Scientific) to align reads to the genome reference (HG19) and to generate run metrics, including chip loading efficiency and total read counts and quality. Following data analysis, annotation of single-nucleotide variants, insertions, deletions, and splice site alterations was performed by the Ion Reporter Server System (Life Technologies) and Ingenuity Variant Analysis (Qiagen).

### 2.5. Sanger Sequencing

The variant of interest was confirmed by Sanger sequencing of amplified PCR products.

## 3. Results

### 3.1. Clinical Presentation

#### 3.1.1. Case 1

**Figure 1:**
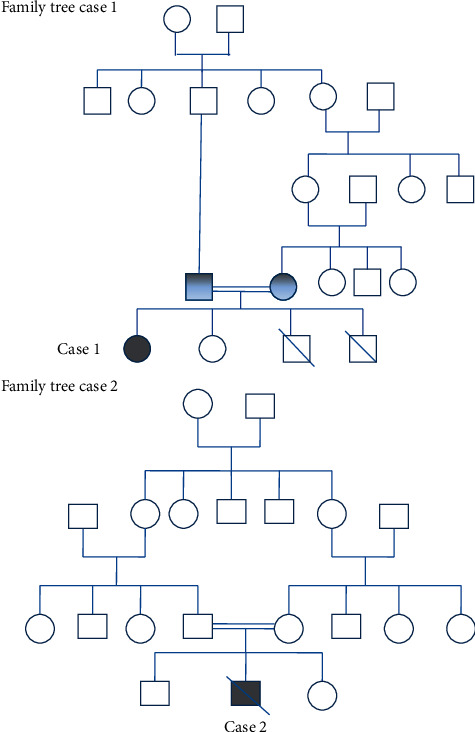
Pedigrees of families with RAG2 mutation: case 1 and case 2. Squares: male subjects; circles: female subjects; black filled symbols: patients with mutation; grey filled symbols: mutation carriers; crossed-out symbols: deceased subjects.

The patient was a newborn female from third-degree consanguineous marriage (Figure 1). She had a familial history of death of siblings. The first brother had recurrent respiratory infections with chronic diarrhea and died at the age of 4 months without any diagnosis. The second brother was admitted to the intensive care unit for severe respiratory infection at six months of age and died three days after admission. The laboratory workup showed lymphopenia, and a SCID was suspected. Considering her family history, our patient was not vaccinated and an immunological workup was done at the age of 20 days (Table 1).

Therefore, the diagnosis of a SCID T-B-NK+ was established. She was put on trimethoprim-sulfamethoxazole as a prophylaxis and on immunoglobulin replacement given every three weeks. She was free from infection until the age of one and a half months, when she developed oral thrush, treated by fluconazole. At the age of 2 months, she developed an axillary lymphadenitis which was treated successfully with ceftazidime and amikacine, given for ten days. At three months of age, the infant underwent successful haploidentical hematopoietic stem cell transplantation with good outcome. Four months posttransplant, there was a good immunoglobulin production with a 100% stable lymphoid chimerism. On the last follow-up, the patient was 5 years old, infection-free with no signs of GVHD, and completely vaccinated.

#### 3.1.2. Case 2

The patient was a four-month-old boy from first-degree consanguineous marriage (Figure 1). He presented by the age of two months chronic diarrhea, recurrent respiratory tract infections, oral thrush, locoregional BCGitis, and failure to thrive. The laboratory workup showed a profound lymphopenia and an absent thymus shadow in the chest X-ray. A diagnosis of SCID was suspected, and an immunological workup was done (Table 2).

Therefore, the diagnosis of a SCID was established. He was on antituberculosis to treat the BCGitis (rifampicine, isonhiazid, and ethambutol) and antifungal (fluconazole). He received also trimethoprim-sulfamethoxazole as a prophylaxis and immunoglobulin replacement every three weeks. Each infectious episode was treated actively. Due to a lack of hematopoietic stem cell transplantation, he died by the age of 1 year and a half.

### 3.2. Genetic Study

The mutation was detected with the complete RAG2 gene sequencing using the Personal Genome Material and the Ion Reporter System and Ingenuity Software and then confirmed by direct target Sanger sequencing for each patient.

**Figure 2:**
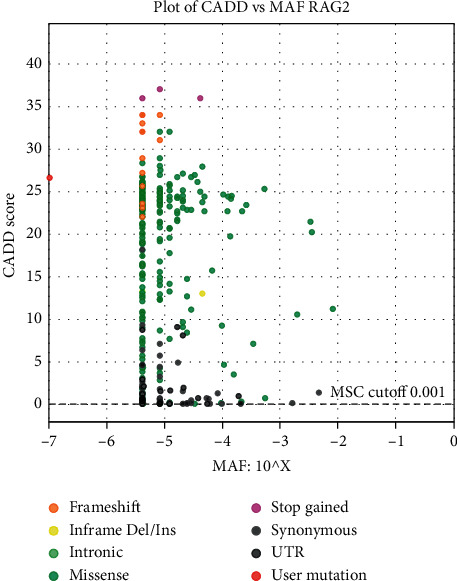
CADD vs. MAF plot of RAG2 by PopViz. The vertical and horizontal axes show the combined annotation-dependent depletion (CADD) and the minor allele frequency (MAF) scores, respectively. The red dot is the c.826 G > A variant and the other dots are the other variants for pathogenic RAG2 mutations (frameshift mutations, inframe Del/Ins mutations, intronic mutation, missense mutations, etc.) reported. The MAF score is −7 and the CADD score is 26.6, which indicates that the new mutation c.826 G > A is probably deleterious.

The two patients carried a variant in the RAG2 gene, c.826G > A (p.Gly276Ser), in a homozygous state. Moreover, for the first case, familial segregation was realized and exhibited the same variant in the RAG2 gene, c.826G > A (p.Gly276Ser), in a heterozygous state in both parents. It is located in a highly conserved nucleotide and amino acid position, with small physicochemical differences between the exchanged amino acids (Alamut v.2.7.1). Software analyses: SIFT, polyphen-2, and Grantham indicated that this variant is highly damaging with a score of 0.0, 1.0, and 56.0, respectively; this was also predicated to damage in the software PopViz (Figure 2), with the MAF score of −7 and the CADD score of 26.6. This variant c.826G > A (p.Gly276Ser) is considered a new mutation that could cause SCID. To date, this variant is not described in the Exome Aggregation Consortium, Exome Sequencing Project, or the 1000 Genomes Browser. To the best of our knowledge, this is the first study, which has revealed such a variant. It is classified as a variant of uncertain significance (class 3) according to the recommendations of the American College of Medical Genetics and Genomics (ACMG).

## 4. Discussion

The RAG1 and RAG2 proteins constitute a recombinase complex, which starts the VDJ somatic recombination by cleaving double-stranded DNA at special sequences, known as recombination signal sequences (RSSs), flanking the variable , diversity , and joining gene exons encoding the genes of immunoglobulins (Ig) and T-cell receptors. This somatic recombination generates a diverse repertoire of B cells and T cells [14, 15]. Mutations in RAG1/2 genes lead to loss or reduction of V(D) J recombination, consequently a blockade in B- and T-cell development, which is referred to as severe combined immunodeficiency (SCID). Our two patients expressed a typical clinical SCID phenotype with opportunistic infections and especially in the second case which was very symptomatic. The two patients had a profound lymphopenia with a very low number of CD3 T cells. The software analyses (SIFT, polyphen-2, Grantham, and PopViz) indicate that this novel mutation c.826G > A in RAG 2 gene is deleterious and then probably responsible for SCID. This was also supported by the familial segregation which exhibited the same variants in the heterozygous state.

## 5. Conclusion

We report in this article a newly discovered mutation in exon 2 of the RAG2 gene c.826G > A in a homozygous state. These results indicate that the AmpliSeq libraries, PGM and the Ion Reporter Sever System- based NGS approach is extremely efficient, fast, and cheap. This approach could lead to a better patients management and genetic counseling for relatives at risk.

## Figures and Tables

**Table 1:** Immunological workup.

(1) HIV serology = negative
(2) CBC: Hb: 18.3 g/dl (8.00–17.6 g/dl), PNN: 1976 c/mm^3^ (1,000–9,500 c/mm^3^), lymphocytes: 832 c/mm^3^ (2,000–17,000 c/mm^3^), and Plts: 383,000 c/mm^3^ (170,000–500,000 c/mm^3^)
(3) Immunoglobulin dosage: IgG: 6.20 g/l, IgM: 0.20 g/l, and IgA: 0.17 g/l
(4) Lymphocyte subpopulation: lymphocytes: 520 c/mm^3^, CD3: 26 c/mm^3^ (2,500–5,900 c/mm^3^), CD4: 10 c/mm^3^ (1,400–4,300 c/mm^3^), CD8: 16 c/mm^3^ (500–1,700 c/mm^3^), CD19: 1 c/mm^3^ (300–3,000 c/mm^3^), and CD16-56: 493 c/mm^3^ (160–950 c/mm^3^)

Note: this table shows laboratory results at the time of admission. All normal ranges cited here are adapted to the age [12, 13].

**Table 2:** Immunological workup.

(1) HIV serology = negative
(2) CBC: Hb: 11 g/dl (8.6–13.7 g/dl), PNN: 1,500 c/mm^3^ (1,500–6,900 c/mm^3^), lymphocytes: 680 c/mm^3^ (3,900–9,000 c/mm^3^), and Plts: 114,300 c/mm^3^ (175,000–500,000 c/mm^3^)
(3) Immunoglobulin dosage: IgG: 0.4 g/l (2.9–5.5 g/l), IgM: 0.04 g/l (0.3–0.85 g/l), IgA: 0.07 g/l (0.2–0.62 g/l), and IgE: 0.11 UI/l
(4) Lymphocyte subpopulation: lymphocytes: 680 c/mm^3^ (3,900–9,000 c/mm^3^), CD3: 62 c/mm^3^ (2,500–5,600 c/mm^3^), CD4: 10 c/mm^3^ (1,800–4,000 c/mm^3^), CD8: 30 c/mm3 (590–1,600 c/mm^3^), CD19: 10 c/mm^3^ (430–3,000 c/mm^3^), and CD16-56: 600 c/mm3 (170–830 c/mm^3^)

Note: this table shows laboratory results at the time of admission. All normal ranges cited here are adapted to the age [12, 13].
